# SHARP hypofractionated stereotactic radiotherapy is well tolerated in prostate cancer: Toxicity and quality of life assessment

**DOI:** 10.1007/s00066-016-0971-2

**Published:** 2016-05-25

**Authors:** Monika Rucinska, Anna Kieszkowska-Grudny, Sergiusz Nawrocki

**Affiliations:** Department of Oncology, University of Warmia and Mazury in Olsztyn, Olsztyn, Poland; Department of Radiation Oncology, Independent Public Health Care Facility of the Ministry of the Interior with Warmia and Mazury Oncology Centre in Olsztyn, Wojska Polskiego 37, 10-228 Olsztyn, Poland; Polish Association of Cognitive and Behavioural Therapy, Minds of Hope, Warsaw, Poland; Department of Oncology and Radiotherapy, Medical University of Silesia, Katowice, Poland

**Keywords:** Hypofractionated stereotactic radiotherapy, Quality of Life, Rectum, Bladder, Organs of risk, Hypofraktionierte stereotaktische Radiotherapie, Lebensqualität, Rektum, Harnblase, Risikoorgane

## Abstract

**Background:**

Quality of life (QoL) is one of the most significant issues in prostate cancer treatment decisions. This study aimed to investigate the toxicity of hypofractionated stereotactic radiotherapy (SBRT) and QoL after treatment in localized prostate cancer patients.

**Materials and methods:**

A prospective single-center clinical study was performed in low- and intermediate-risk prostate cancer patients. Patients received 33.5 Gy in 5 fractions (SHARP regimen). Acute and late toxicity was assessed according to RTOG/EORTC score. Patients filled out EORTC QLQ-C30 and prostate cancer-specific QLQ-PR25 questionnaires.

**Results:**

The analysis included 68 prostate cancer patients (55–83 years, median 73) with clinical stage T1c-T2cN0M0, median combined Gleason score of 6 (3–8), and median prostate-specific antigen (PSA) level of 10 ng/mL (4–20 ng/mL). Neoadjuvant androgen deprivation therapy was given to 52 patients (76.5 %), and stopped in 31 patients (45.5 %) after 6 months; in 21 patients (31 %) after 2–3 years. Average and median follow-up was 24 months (18–45). Median nadir PSA level was 0.03 ng/mL for all patients and 0.6 ng/mL for patients without hormone treatment. No patients had PSA failure. There were no acute grade IV toxicities. One patient (1.5 %) developed grade III and 24 patients (35.3 %) grade II acute bladder toxicity. No one developed grade III and 7 patients (10.3 %) grade II acute rectal toxicity. No grade III or IV late gastrointestinal or genitourinary toxicities were reported. Grade II late urinary symptoms were observed in 8 patients (11.8 %) and gastrointestinal symptoms in 3 patients (4.4 %). Global health status/QoL was good and improved during the observational period.

**Conclusion:**

SBRT for prostate cancer patients is a well-tolerated treatment in terms of toxicity and QoL, has no negative impact on functioning and everyday life, with the important benefit of a short treatment period. However, long-term follow-up data are needed.

## Zusammenfassung

**Hintergrund:**

Die Lebensqualität (QoL) ist zu einem der wichtigsten Schwerpunkte bei der Wahl der Prostatakarzinombehandlung geworden. Das Thema dieser Studie war die Untersuchung der Toxizität der hypofraktionierten stereotaktischen Radiotherapie (SBRT) und der QoL nach Behandlung des lokal begrenzten Prostatakarzinoms.

**Materialien und Methoden:**

Die prospektive, monozentrische, klinische Studie wurde bei Prostatakarzinompatienten mit niedrigem bis mittlerem Risiko durchgeführt. Die Patienten erhielten 33,5 Gy in 5 Fraktionen (SHARP-Behandlungsschema). Akute und späte Toxizität wurden nach den Kriterien des RTOG/EORTC-Scores klassifiziert. Die Patienten füllten das EORTC-QLQ-C30- und das prostatakarzinomspezifische QLQ-PR25-Formular aus.

**Ergebnisse:**

Die Analyse umfasste 68 Prostatakarzinompatienten (medianes Alter 73, Spanne 55–83 Jahre) im klinischen Staging T1c–T2cN0M0, mit einem medianen Gleason-Score von 6 (Spanne 3–8) und einem medianen PSA-Wert (prostataspezifisches Antigen) von 10 ng/ml (Spanne 4–20 ng/ml). Eine neoadjuvante Androgendeprivationstherapie erhielten 52 Patienten (76,5 %); die Hormontherapie beendet 31 Patienten (45,5 %) nach 6 Monaten und 21 Patienten (31 %) nach 2–3 Jahren. Das durchschnittliche und mediane Follow-up dauerte 24 Monate (Spanne 18–45). Der mediane PSA-Nadir betrug 0,03 ng/ml für alle Patienten und 0,6 ng/ml für Patienten ohne Hormontherapie. PSA-Versagen und akute Grad-IV-Toxizitäten traten nicht auf. Ein Patient (1,5 %) hatte eine Grad-III- und 24 Patienten (35,3 %) eine Grad-II-Harnblasentoxizität, kein Patient eine Grad-III- und 7 Patienten (10,3 %) eine akute Grad-II-Rektumtoxizität. Späte gastrointestinale oder urogenitale Toxizitäten III. oder IV. Grades wurden nicht berichtet. Bei 8 Patienten (11,8 %) traten späte Miktionsbeschwerden II. Grades und bei 3 Patienten (4,4 %) gastrointestinale Symptome auf. Globaler Gesundheitsstatus/QoL war gut und besserte sich in der untersuchten Zeit.

**Schlussfolgerung:**

SBRT ist beim Prostatakarzinom eine gut tolerierte Behandlung hinsichtlich Toxizität und QoL, ohne negativen Einfluss auf das Alltagsleben und dem wichtigen Vorteil einer kurzen Behandlungszeit. Langfristige Follow-up-Untersuchungen müssen noch folgen.

Prostate cancer is the second most common solid tumor in men worldwide [1]. The standard treatment for early-stage prostate cancer is surgery or radiotherapy. Radical prostatectomy is an option for men with a life expectancy of at least 10 years. Radiotherapy is a reasonable alternative to surgery. Observational data and retrospective analyses suggest that the results of surgical treatment and radiation therapy are similar in patients with localized prostate cancer. The disease-specific survival rate is 98 % for patients after radical prostatectomy and 97 % for patients after external beam radiotherapy (*p* = 0.543) [2]. In the case of clinically localized, very low- and low-risk prostate cancer, active surveillance (“wait and see”) is also an option.

The goal of radiotherapy is to deliver an adequate dose of radiation to the target, in this case the prostate, with an appropriate margin and while minimizing the dose to normal tissues (in the rectum, bladder, bulb of penis, and femoral heads). Three-dimensional conformal radiation therapy (3D-CRT) has replaced the old two-dimensional technique and has been the standard treatment for prostate cancer patients for years. Dose escalation with intensity-modulated radiation therapy (IMRT) resulted in improved cancer control in comparison to 3D-CRT, without increased toxicity [3]. IMRT has now been established as the standard external beam modality in low-risk prostate cancer [4–6]. Image-guided radiotherapy (IGRT) is used to reduce the volume of irradiated normal tissue [7, 8]. Recent data suggest that hypofractionated radiotherapy (2.5–3.1 Gy per fraction) results in high local control of prostate cancer with acceptable toxicity [9–11]. Stereotactic body radiation therapy (SBRT) is an extreme form of hypofractionation that uses several high-dose fractions (6–7 Gy). The first publications on hypofractionation in the treatment of prostate cancer patients came out in the early 1990s [12, 13]. For intermediate-risk patients, androgen deprivation therapy is recommended, and should start 6 months before external beam radiotherapy [14].

The prognosis for most prostate cancer patients, particularly those in an early stage and independent of treatment options, is very good. For prostate cancer survivors, quality of life (QoL) is a very important factor and it has become one of the most significant issues in prostate cancer treatment decisions.

The objective of this study was to investigate the effectiveness and safety of hypofractionated SBRT for localized prostate cancer, as well as patients’ QoL after treatment.

## Materials and methods

A prospective single-center clinical study was performed in low- and intermediate-risk localized prostate cancer patients (according to the National Comprehensive Cancer Network, NCCN). All patients underwent thoracic X-ray, abdominal ultrasonography, pelvic magnetic resonance (MR), and bone scintigraphy.

### Treatment planning

Patients were treated according to the special protocol prepared for this study. Patients were placed on a diet designed to minimize gas production, without milk products, fresh fruits, and vegetables, 14 days before planning and during the entire treatment period. We used three fiducial markers (soft tissue gold markers; Civco, Coralville, IA, USA) for daily image-guided positioning. The patients were in the supine position, immobilized by a vacuum mattress (BlueBAG; Medical Intelligence, Klongtoey, Bangkok), and were set up with four tattoos. Computed tomography (CT) and MR images (acquired at least 14 days after fiducial placement) were used in treatment planning. Slice thicknesses for planning were 1.5 mm for CT and 3 mm for MRI. Planning (and treatment) was carried out with a filled bladder (200–300 mL) and empty rectum. According to International Commission on Radiation Units and Measurements (ICRU) reports 50 and 62 [15, 16], the clinical target volume (CTV) included the prostate and the proximal part of the seminal vesicles (about 1 cm) with a margin of 3 mm (2 mm from the rectum), and the planning target volume (PTV) was equal to the CTV but expanded by a 2 mm isotropic margin. Step-and-shoot IMRT plans were made. According to ICRU report 83 [17], 95 % of the PTV should receive at least 98 % of the prescription dose.

### Treatment

All patients were treated with 15-MV X‑rays using a Primus accelerator (Siemens, Berlin, Germany). The positions of the prostate, rectum, and bladder were visualized daily with MV cone beam CT and portal images. The volumes of rectum and bladder were checked and corrected if necessary. There were daily positioning corrections of the patient with reference to position of the markers. It was necessary to avoid underdosage in the PTV and too high doses to organs of risk [18]. The patients received 33.5 Gy in 5 fractions (6.7 Gy per fraction), similarly to those enrolled in the stereotactic hypofractionated accurate radiotherapy of the prostate (SHARP) trial [19]. Patients were treated twice weekly for a median of 15 days. The radiation dose of 33.5 Gy in 5 fractions was equivalent to the conventional dose of 78 Gy in 39 fractions of 2 Gy each; the α/β ratio for prostate cancer was estimated to be around 1.4–1.5 Gy [9, 20, 21]. The α/β ratio for acute effects in the bladder and rectum is about 10 Gy; thus, the acute effects equivalent dose in this hypofractionated regimen was 46.6 Gy. The α/β ratio for late complications in the rectum is 3 Gy [22]. Taking this into account, the late rectal reactions dose was equivalent to the dose of 65 Gy in 2‑Gy fraction regimens.

### Follow-up

Prostate-specific antigen (PSA) levels were obtained before treatment and every 3 months. Eventual failure was defined as nadir plus 2 ng/mL according to the Phoenix definition of PSA failure [23, 24].

Acute and late toxicity assessments according to the Radiation Therapy Oncology Group and the European Organization for Research and Treatment of Cancer (RTOG/EORTC) score were carried out during radiotherapy, 1 and 3 months after the end of treatment, and then every 3 months. QoL evaluations were done thrice: at least 9 months after radiotherapy and then every 9 months. The patients filled out the EORTC QLQ-C30 and the prostate cancer-specific QLQ-PR25 questionnaires.

The study protocol was approved by the local ethics review board of the University of Warmia and Mazury in Olsztyn, Poland. All patients submitted a signed consent form.

We used demographic frequencies and descriptive statistics in the analysis, as well as a Students’ *t*-test and a general linear model to measure the mean differences between time assessments.

## Results

Patients were considered eligible for inclusion in this study if they had previously untreated, histologically confirmed adenocarcinoma of the prostate. The analysis included 68 men (age 55–83 years, mean 72.5 years, median 73 years) treated between August 2011 and September 2013 at the Department of Radiation Oncology of the Independent Public Health Care Facility of the Ministry of the Interior with Warmia and Mazury Oncology Centre in Olsztyn, Poland. The clinical stage of prostate cancer was T1c-T2cN0M0, the combined Gleason score was 3–8 (mean and median 6), PSA level was 4–20 ng/mL (mean 10.9 ng/mL, median 10 ng/mL). Neoadjuvant androgen deprivation therapy beginning a maximum of 6 months before radiotherapy was given to 52 patients (76.5 %). Hormonal therapy was stopped after 6 months in 31 patients (45.5 %). For 21 patients (31 %), androgen blockade was planned for 2 to 3 years; this was stopped during follow-up in all patients except two. Of the patients who did not receive androgen deprivation therapy, 16  (23.5 %) had a median pretreatment PSA level of 7.53 ng/mL (mean 8.55 ng/mL; Tab. 1).

**Tab. 1:** Patients’ characteristics

		Number (total *N* = 68)	%
*Age (years)*	55–83 (median 73, mean 72.5)		
	≤ 65	10	15
	> 65	58	85
*TNM*			
	T1cN0M0	6	9
	T2aN0M0	15	22
	T2bN0M0	19	28
	T2cN0M0	28	41
*Gleason score*	3–8 (median 6, mean 6)		
	3	2	3
	5	21	31
	6	14	20.5
	7	29	42.5
	8	2	3
*PSA (ng/mL)*	4–20 (median 10, mean 10.9)		
	≤ 10	35	51
	> 10	33	49
*Risk group*			
Low-risk		7	10
Intermediate-risk		61	90
*Hormone therapy*			
	Without	16	23.5
	6 months	31	45.5
	2–3 years	21	31

*PSA* prostate-specific antigen

Preparation for radiotherapy, according to the protocol prepared for this study, did not cause a problem for the patients. The daily setup took approximately 50 minutes; radiotherapy was delivered in about 6 minutes per fraction.

All patients completed the treatment. The average and median follow-up was 24 months. The follow-up was stopped after 9 months for one patient and after 12 months for another because of other illnesses; all other patients had a follow-up visit at least 18 months after the end of treatment. No patients died during the observation period. One patient developed sigmoid colon cancer 24 months after the end of radiotherapy.

The PSA response was favorable. The median 3‑month posttreatment PSA levels were 0.08 ng/mL for all patients and 2.8 ng/mL for those who did not receive androgen deprivation therapy. Twelve months after the end of radiotherapy, the median PSA levels were 0.06 ng/mL for all patients, 1.6 ng/mL for those who did not receive androgen deprivation therapy, and 0.04 ng/mL for patients who underwent 6 months of hormone therapy. Twenty-four months after the end of radiotherapy, the median PSA levels were 0.1 ng/mL for all patients, 0.4 ng/mL for those who did not receive androgen deprivation therapy, and 0.1 ng/mL for patients who underwent 6 months of hormone therapy. The median nadir PSA levels were 0.03 ng/mL (mean 0.22 ng/mL) for all patients and 0.6 ng/mL (mean 0.7 ng/mL) for patients without hormone treatment. At the median 24-month follow-up, there were no patients with PSA failure (nadir plus 2 ng/mL). After 6 months of androgen deprivation, the PSA level of two patients increased by over 1 ng/mL from the nadir during the observation period. More than two thirds of the patients (75 %) showed stable or decreased levels of PSA.

**Fig. 1:**
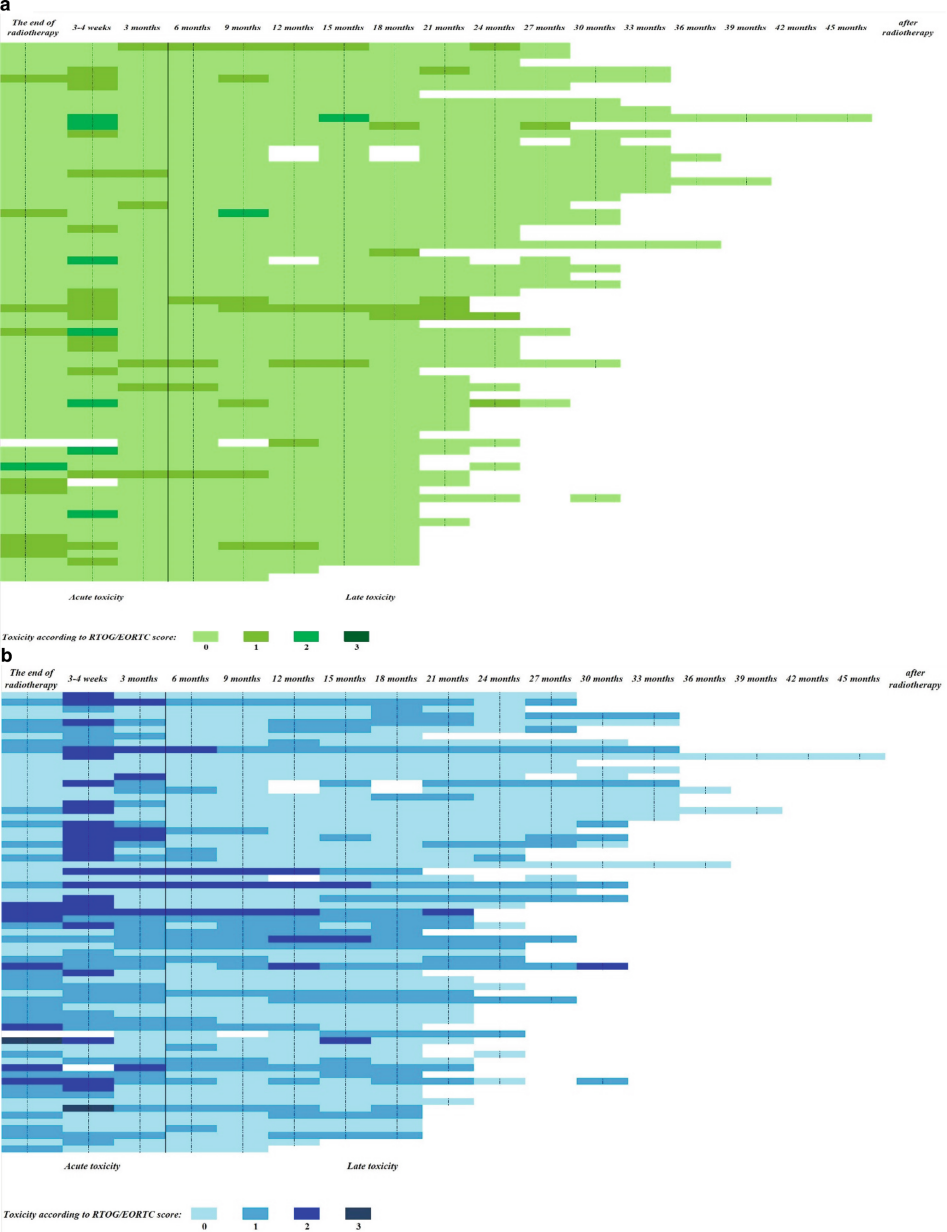
Acute and late gastrointestinal (**a**) and genitourinary (**b**) toxicities according to Radiation Therapy Oncology Group/European Organization for Research and Treatment of Cancer (RTOG/EORTC) score

Patients’ tolerance of the treatment was good. There were no acute grade IV toxicities. Most of the patients (67.6 %) developed mild and moderate (grade I or II) acute genitourinary and/or gastrointestinal toxicities: 24 patients (35.3 %) developed grade II acute bladder toxicity and 7 patients (10.3 %) grade II acute rectum toxicity. Only one patient (1.5 %) had grade III genitourinary toxicities; no patients demonstrated grade III acute gastrointestinal toxicities. No grade III or higher late genitourinary or gastrointestinal or toxicities were reported. More than one third of all patients (38 %) did not develop any late radiation-related complications. More than 3 months after radiotherapy, 8 patients (11.8 %) had moderate (grade II) urinary symptoms. No urinary incontinence was observed. Only 3 patients (4.4 %) presented moderate (grade II) gastrointestinal symptoms more than 3 months after the treatment had ended (Tab. 2, Fig. 1).

**Tab. 2:** Acute and late gastrointestinal and genitourinary toxicities according to RTOG/EORTC score

	Genitourinary toxicities		Gastrointestinal toxicities	
	Number	%	Number	%
*Acute toxicities*				
0	21	30.9	43	63.2
Grade I	22	32.3	18	26.5
Grade II	24	35.3	7	10.3
Grade III	1	1.5	0	0
Grade IV	0	0	0	0
*Late toxicities*				
0	32	47	53	78
Grade I	28	41.2	12	17.6
Grade II	8	11.8	3	4.4
Grade III	0	0	0	0
Grade IV	0	0	0	0

*RTOG/EORTC* Radiation Therapy Oncology Group/European Organization for Research and Treatment of Cancer

**Fig. 2:**
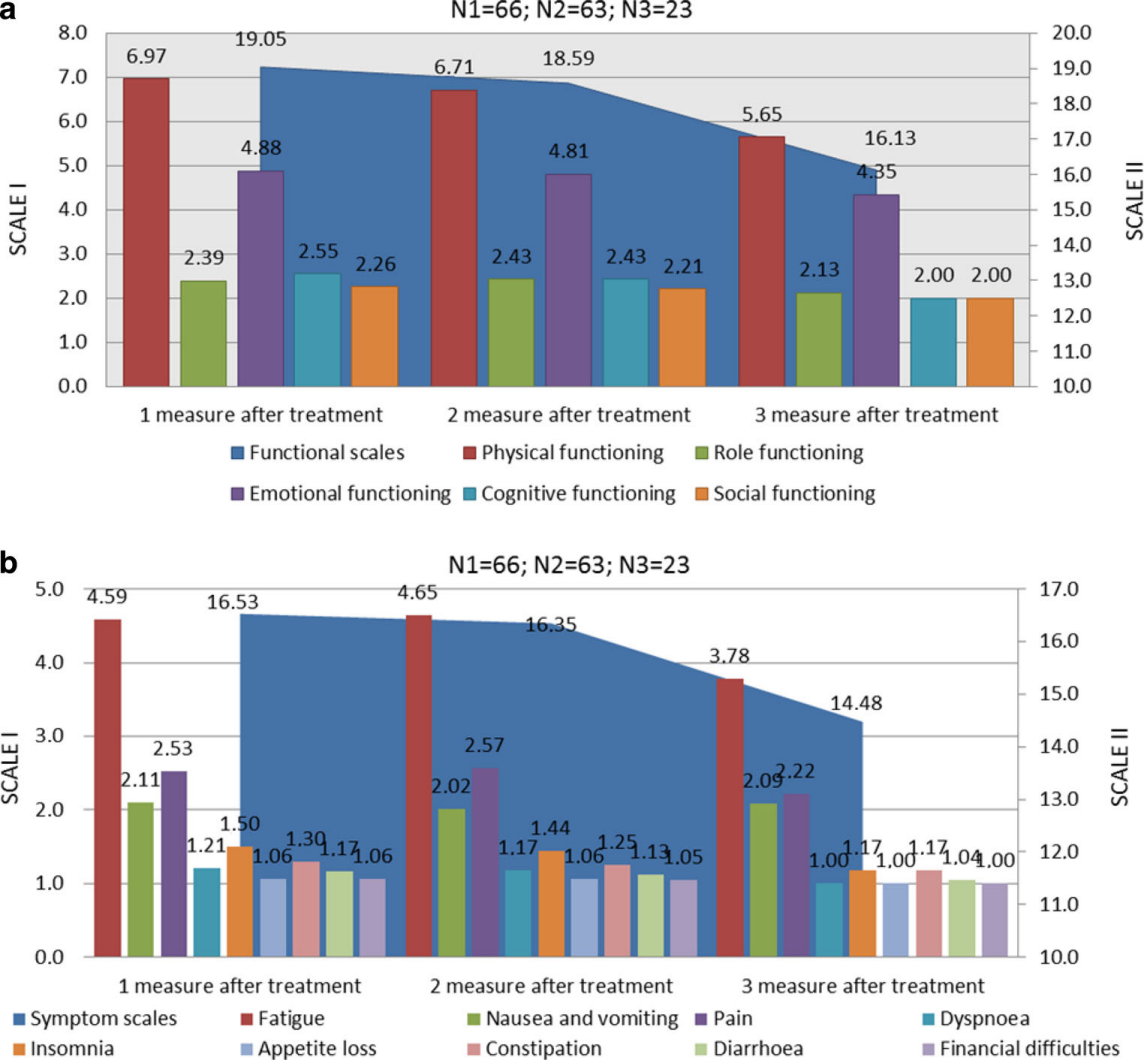
Mean results of quality of life functional scales (**a**) and assessment of symptoms (**b**) in three measuring points of observation (in median 9, 21 and 30 months after end of radiotherapy). **a** Scale I (*left site*) presents subscales of QoL: physical functioning, emotional functioning, cognitive functioning, role functioning and social functioning; scale II (*right site*) presents summary results for all functional scales. Lower results indicate better functioning. **b** Scale I (*left site*) presents subscales of each symptoms: fatigue, nausea and vomiting, pain, dyspnea, insomnia, loss of appetite, constipation, diarrhea, financial difficulties; scale II (*right site*) presents summary results for all symptoms. Lower results indicate fewer symptoms

Evaluations of QoL were done thrice: at 9 months after radiotherapy, T1 for all patients except 2 (because of difficulty in coming for evaluation due to other diseases); at 21 months after the end of radiotherapy (63 patients), T2; and at 30 months after the end of radiotherapy (23 patients), T3. The global health status (GHS)/QoL improved during the observational period (mean, M; median, Me; standard deviation, SD): MQoL1 = 10.09 (*N* = 66; MeQoL1 = 10.00; SD = 1.44), MQoL2 = 10.51 (*N* = 63; MeQoL2 = 10.00; SD = 1.70), and MQoL3 = 11.30 (*N* = 23; MeQoL3 = 12.00; SD = 1.18), 9, 21 and 30 months after radiotherapy, respectively. The biggest functional issues for prostate cancer patients were physical and emotional functioning. In turn, the dominant symptom was fatigue, which decreased during the observation period (Fig. 2). We observed significant or close to significant effects at three measurement points for specific functional subscales of QoL and general symptoms connected with cancer and oncologic treatment: physical functioning (F[2.44] = 3.731; *p* < 0.05; eta2 = 0.145), emotional functioning (F[2.44] = 3.074; *p* = 0.056; eta2 = 0.123), cognitive functioning (F[2.44] = 5.714 with Greenhouse-Geisser correction, GGc; *p* < 0.01; eta2 = 0.206), social functioning (F[2.44] = 2.997 with GGc; *p* = 0.068; eta2 = 0.120), and general functional QoL (F[2.44] = 5.747 with GGc; *p* < 0.01; eta2 = 0.207; Tab. 3). Among 25 prostate cancer-specific medical issues, five variances were reported to be significant: “urinating frequently at night” (F[2.42] = 5.370 with GGc; *p* < 0.05; eta2 = 0.204), “hurrying to get to the toilet” (F[2.42] = 4.177; *p* < 0.05; eta2 = 0.166), “not getting enough sleep because of the need to get up frequently at night to urinate” (F[2.42] = 3.249 with GGc; *p* < 0.05; eta2 = 0.134), “difficulty in going out of the house because of the need to be near a toilet” (F[2.42] = 5.332 with GGc; *p* < 0.05; eta2 = 0.202), and “hot flushes” (F[2.42] = 5.332 with GGc; *p* < 0.05; eta2 = 0.202).

**Tab. 3:** Pairwise comparison between specific quality of life (QoL) subscales at T1, T2, and T3 measures (only significant or close to significant effects are presented)

1st factor at specific time	2nd factor at specific time	Mean difference	SD	*P*-value	95 % CI for mean difference	
					Lower limit	Upper limit
Physical functioning T3	Physical functioning T1	−1.130^*^	0.418	0.039	−2.212	−0.049
	Physical functioning T2	−1.000^*^	0.367	0.036	−1.947	−0.053
Emotional functioning T1	Emotional functioning T2	−0.522^*^	0.176	0.022	−0.977	−0.066
	Emotional functioning T3	0.087	0.288	0.987	−0.656	0.830
Cognitive functioning T3	Cognitive functioning T1	−0.478^*^	0.152	0.014	−0.872	−0.085
	Cognitive functioning T2	−0.565^*^	0.176	0.012	−1.020	−0.111
Social functioning T1	Social functioning T2	−0.043	0.194	0.995	−0.544	0.457
	Social functioning T3	0.391	0.163	0.074	−0.030	0.813
Functional scales T3	Functional scales T1	−2.435^*^	0.757	0.012	−4.389	−0.480
	Functional scales T2	−2.913^*^	0.994	0.023	−5.481	−0.345
Insomnia T1	Insomnia T2	0.087	0.188	0.956	−0.398	0.572
	Insomnia T3	0.391	0.163	0.074	−0.030	0.813
Constipation T1	Constipation T2	0.174	0.120	0.411	−0.136	0.484
	Constipation T3	0.304^*^	0.117	0.047	0.003	0.605
Symptoms scale T1	Symptoms scale T2	0.391	0.821	0.953	−1.731	2.514
	Symptoms scale T3	1.739^*^	0.488	0.005	0.479	3.000

Based on the least squares means

*CI* confidence interval, *SD* standard deviation, T1, T2, T3 times of evaluation (9, 21 and 30 months after radiotherapy)

^*^The difference in a significant level 0.05

## Discussion

Hypofractionated SBRT is a novel technique for the treatment of early-stage prostate cancer. Preliminary data have shown that this approach leads to successful tumor control without increasing complications [25–27].

Acute urinary and rectal toxicities during and after SBRT are not higher than those for 3D-CRT and IMRT. Collins et al. [12] reported the good outcome of 232 patients with both early and advanced disease who were treated with 36 Gy in 6 fractions. Soete et al. [28] reported no grade III or IV acute toxicity among 36 prostate cancer patients treated with 56 Gy in 16 fractions. King et al. [26], on behalf of the Multi-institutional Consortium of Prospective Trials, presented an analysis of 864 patients from phase II clinical trials of SBRT (median dose of 36.25 Gy in 4–5 fractions) for localized prostate cancer (median follow-up of 3 years; 194 patients remained evaluable at 5 years). Some problems with the bladder and rectum were observed within the first 3 months after SBRT, but the conditions returned to baseline status or better within 6 months.

In the current study, 42 % of patients showed no treatment-related reaction during the radiotherapy schedules. There were no grade III or IV toxicities in the rectum, and only one patient (1.5 %) developed grade III toxicity in the bladder. Similarly, in the SHARP study [19], only one acute grade III genitourinary toxicity during treatment was reported (2.5 %). In the present study, 31 % of the patients had no bladder reaction, compared with up to 49 % in the SHARP trial. Furthermore, in the SHARP trial, 61 % of the patients had no acute (during and 1 month after the end of treatment) gastrointestinal toxicity, whereas this value was 63.5 % in our study. The late toxicity result in our study was similar to that in the SHARP trial: no grade III or higher late gastrointestinal or genitourinary toxicities were reported. No late genitourinary toxicity was developed by 55 % of the patients in the SHARP trial and 47 % in our study. Late gastrointestinal toxicity was even rarer, with 62.5 % of the patients in the SHARP trial and 78 % in our study not developing such toxicity. There were no grade III rectal reactions either in the SHARP trial or in our study. The King et al. series [29], with a median follow-up of 2.7 years, reported no grade III or higher rectal toxicity and no grade IV urinary toxicity; only 3.5 % of the patients developed grade III urinary toxicity (36.25 Gy in 5 fractions).

Yarbro and Ferrans [30] demonstrated that radiotherapy has little impact on deterioration of QoL. Our research confirms that QoL of prostate cancer patients undergoing SBRT is at a satisfactory level. We observed that the GHS/QoL of the patients was good 9 months after the end of treatment and significantly improved during the following months. Functional aspects, such as physical, emotional, cognitive, social, and general functioning, also improved.

The American Society for Therapeutic Radiation and Oncology (ASTRO) definition of biochemical PSA failure as a surrogate endpoint for recurrence is three consecutive increases in the PSA level after the posttreatment PSA nadir dated at the midpoint between the nadir and the first increase [31]. The RTOG Phoenix definition consists of a PSA level that increases to more than 2.0 ng/mL from the nadir. As a potential surrogate endpoint in clinical trials, the Phoenix definition of PSA failure is a strong correlate of mortality and a predictor of metastatic disease; it is superior to the ASTRO definition [32]. The 2‑year survival rate without PSA failure ranges from 90 to 100 % [33]. Thus far, only one observation of patients treated with SBRT has been performed, with a median follow-up of 5 years [34]: the biochemical progression-free survival rate was 93 % in a cohort of 41 consecutive patients (35 or 36.25 Gy in 5 fractions); the median PSA nadir was 0.3 ng/mL. Madsen et al. [35], who applied the same fractionation schedule, found that the majority of nadirs were less than 1.0 ng/mL. In our study, the median PSA nadir for patients without hormone treatment was 0.6 ng/mL. At a median 24-month follow-up, there were no patients with PSA failure. For 2 patients, the PSA level increased by over 1 ng/mL from the nadir; for 15 patients, the increase was 0.2–1.0 ng/mL from the nadir (25 % of all patients). King et al. [36] observed an increase in PSA level of > 0.2 ng/mL in 16 % of patients at a median 36-month follow-up.

There is more information about the CyberKnife (Accuray, Sunnyvale, CA, USA) than linear accelerator (LINAC) use for SBRT of prostate cancer patients. Our study used a LINAC, and all our results (acute and late toxicities, QoL, and PSA increase and failure) are similar to those obtained with the CyberKnife series.

One of the potential benefits of SBRT is a short treatment period and probably lower costs compared with other advanced techniques. Sher at al. [37] showed that SBRT is a more cost-effective (in terms of radiotherapy, treatment of acute and late toxicities, and quality-adjusted life year) external beam modality than IMRT.

Our data are preliminary, as the number of treated patients is relatively small and follow-up should be longer. In the intermediate risk-group patients treated with hormonal therapy, the potential curative effect of radiotherapy cannot be assessed reliably during hormonal treatment; therefore the efficacy of SBRT in our patients could not be estimated precisely and further follow-up is necessary. Follow-up is planned for 10 years and will be presented in the future. One of the potential shortcomings was lack of the pre-study QoL assessment; however we believe that QoL measured thrice after treatment is informative.

## Conclusions

Hypofractionated stereotactic radiotherapy for low- and intermediate-risk prostate cancer patients is a safe and convenient treatment in terms of its duration, PSA response, toxicity and patients’ QoL assessment in the short term. However, a longer follow-up is needed.
